# Three Dimensional Lower Extremity Musculoskeletal Geometry of the Visible Human Female and Male

**DOI:** 10.1038/s41597-022-01905-2

**Published:** 2023-01-18

**Authors:** Thor E. Andreassen, Donald R. Hume, Landon D. Hamilton, Karen E. Walker, Sean E. Higinbotham, Kevin B. Shelburne

**Affiliations:** University of Denver, Center for Orthopaedic Biomechanics, Denver, CO USA

**Keywords:** Musculoskeletal system, Translational research

## Abstract

Models and simulations of human function impact medicine and medical technology. Particularly, musculoskeletal modeling provides an avenue for insight into the human body, which might not be otherwise possible. However, reaching the ultimate goal of functional multi-scale human models has been slowed by the lack of freely available datasets of anatomical models and geometries. Moreover, female-specific geometries have been neglected with a widespread emphasis on male geometry. To help realize this goal, we have developed and shared complete three-dimensional musculoskeletal geometries extracted from the National Libraries of Medicine Visible Human Female and Male cryosections. Muscle, bone, cartilage, ligament, and fat from the pelvis to the ankle were digitized and exported. These geometries provide a foundation for continued work in human musculoskeletal simulation with high-fidelity deformable tissues that enable a better understanding of normal function and the evaluation of pathologies and treatments. This work is novel as it includes both the male and female Visible Human specimens, outputs at multiple levels of post-processing for maximum data reuse, and is publicly available.

## Background & Summary

Musculoskeletal modeling provides an avenue to gain insight into the human body, which might not be possible otherwise. Models and simulations of human function have had a substantial impact on medicine, medical technology, product development, and education. To highlight some of the many examples, models and simulations have been used to understand tissue pathology and treatment^1,2^, analyse response to impact and car-crashes^3^, develop and test products^4^, create education materials^5^, and build models for entertainment^6^. Unfortunately, the three-dimensional (3D) geometries of the human tissues used to create useful applications are infrequently offered to the public, or may be in a raw form with limited utility^7^. As a result, new applications must start from the beginning with the effortful and time-consuming first step in the process of building 3D tissue geometries. 3D geometries are frequently built from the segmentation of anatomy from cross-sectional images (e.g. from MRI or CT). Although geometries created from the Visible Human Project^8,9^ have been used in numerous research and commercial applications, they are also seldom shared. The few exceptions are example models purpose-built for applications such as car-crash analysis or electromagnetic analysis, focusing on organs and general structures for appendages^7,10^. Groups of tissue geometries were combined to create these models, whereas simulation of musculoskeletal movement requires detailed representation of individual muscle geometries. Furthermore, freely available muscle geometries such as those in OpenSim^11^ are represented by two-dimensional lines of action and frequently contain a collection of muscle representations from various specimens. Moreover, there has been widespread utilization of tissue geometries constructed from the Visible Human Male imaging dataset, mostly neglecting the availability of the Visible Human Female imaging dataset. Our work aimed to produce and publicly share a comprehensive set of individual 3D musculoskeletal geometries of the lower extremities, including both the Visible Human Female and Male, and provide the results in multiple levels of post-processing for maximum data reuse. An inherent consequence of creating 3D geometries from imaging is that raw and smoothed geometries may have some overclosure or overlap^12^. A novel part of this work is that the provided final geometries include no overlap and sufficient smoothing to provide a ready foundation for use in computer modeling and simulation, while staying true to the original source imaging. These geometries provide a starting point for continued work in human musculoskeletal simulation with high-fidelity deformable tissues that may enable a better understanding of normal function and the evaluation of pathologies and treatments.

**Fig. 1:**
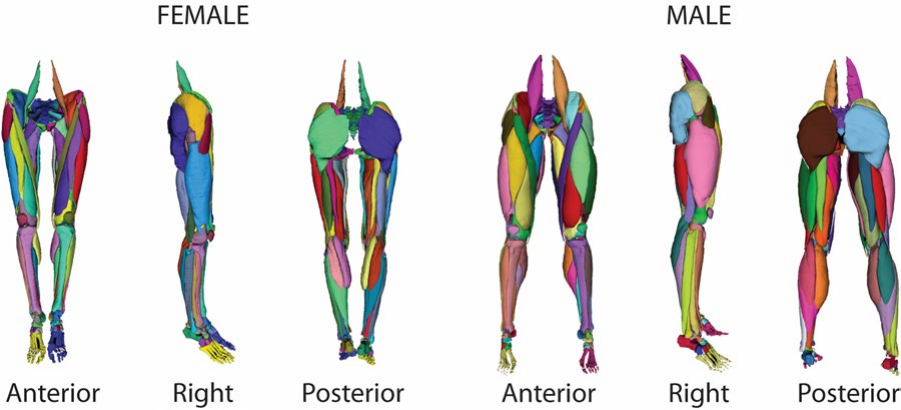
Anterior, lateral, and posterior views of the Female (left) and Male (right) 3D musculoskeletal geometry consisting of 76 muscles, 28 bones, 16 joint cartilage segments, and 8 ligaments.

In summary, 3D musculoskeletal geometries were extracted from the National Libraries of Medicine Visible Human Female and Male cryosections. Muscle, bone, cartilage, ligament, and fat from the pelvis to the ankle were digitized and exported in shareable formats and made available for download. While a substantial amount of published work has been derived from the Visible Human Project, to the authors’ knowledge, this is the first time a large number of 3D geometries are being made available to the public inclusive of both the male and female specimens. In total 260 geometries from the Visible Human Female and Male were extracted from the cryosections consisting of 76 muscles, 28 bones, 16 cartilages, 8 ligaments, and 2 fat geometries per subject (Fig. 1)^13^. The library is available at multiple stages of processing and notably in a final form with no overlap between neighboring structures. This work provides a resource to develop a range of computational models which may allow for more representative models of musculoskeletal mechanics. This library is available online to motivate continued work in multi-scale high-fidelity musculoskeletal modeling and promote reuse and continued development including the addition of new geometries of the musculoskeletal system.

## Methods

**Fig. 2:**
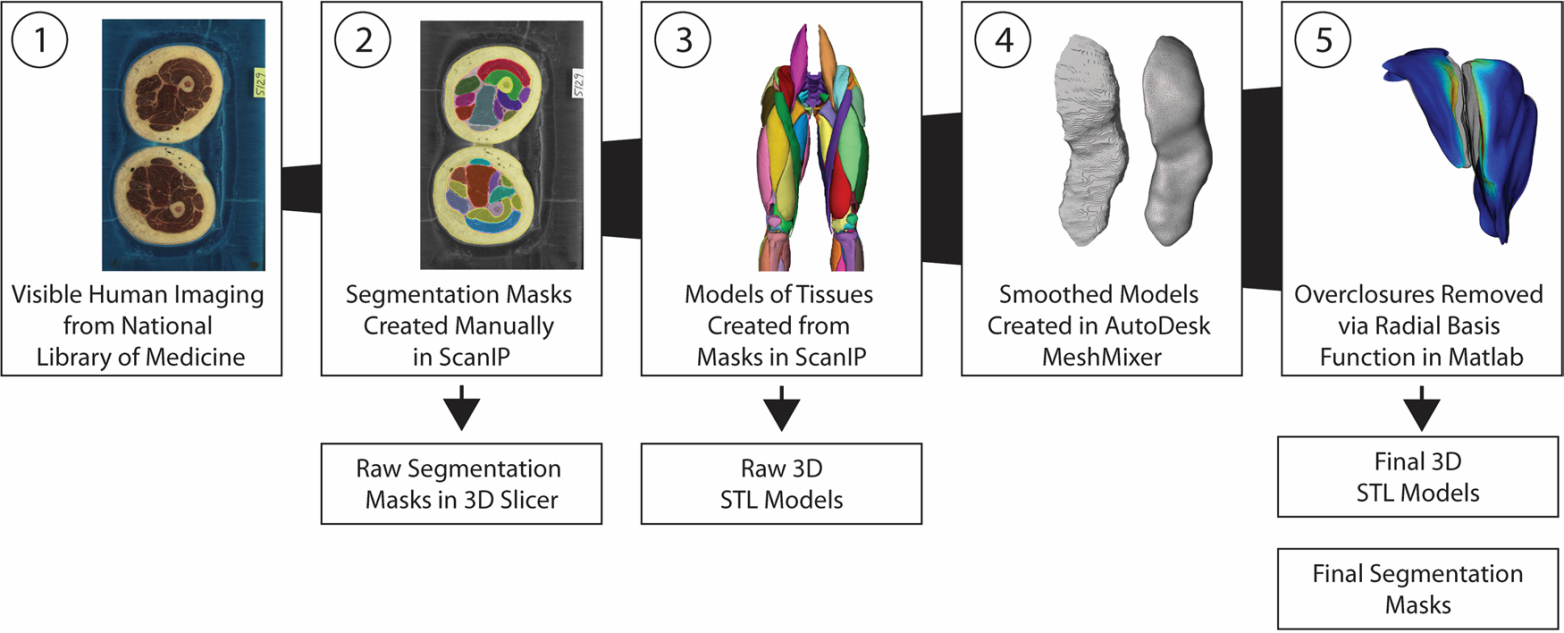
Flowchart showing the primary steps in the model generation process and the publicly shared outputs.

Courtesy of the U.S. National Library of Medicine, images were acquired for both the Visible Human Male (39 M, 71 in., 90 kg, 27.8 BMI) and Female (59 F, 62 in., 88 kg, 36 BMI)^8^. These images consist of digitized axial cryosection color photographs taken at 1 mm intervals in the VHM and 0.33 mm intervals in the VHF. The creation of the 3D models followed these steps (Fig. 2):

1. The cryosection and CT images were downloaded from the National Library of Medicine (https://www.nlm.nih.gov/research/visible/visible_human.html), reduced in size for manageability, and imported into software used for segmenting geometries from medical images (ScanIP v. S-2021.06, Simpleware, Synopsys, Mountain View, CA). Image volumes were cropped to the sternum. Offsets were created using original Cartesian coordinates of images from the original dataset, and manual/automatic alignment registration in non-aligning areas.
2. Segmenting entailed manually selecting geometries in images using ScanIP. The researchers performing the segmentation referenced multiple anatomical sources, primarily Netter^14^ and Fleckenstein *et al*.^15^, and anatomical imaging applications at Radiopedia (www.radiopaedia.org) and AnatomyLearning (www.anatomylearning.com). Primal Pictures (Informa UK Limited, London) software was used as a reference for the Visible Human Male. Segmentation masks from Step 2 were exported from ScanIP as metaimage header (MHD) files to enable alteration or refinement of our digitization process by subsequent users. MHD files were included as the primary files containing the raw segmentation for use with most other commercial software, such as Mimics (Materialise, Belgium), ScanIP, and Amira (ThermoFisher Scientific, Waltham, MA).
3. MHD files were combined in 3D Slicer (v. 4.11.20210226, www.slicer.org) to create nearly raw raster data (NRRD) files, the standard files used in 3D Slicer for segmentation^16^. 3D slicer is a freely available software for reconstruction of three-dimensional geometry from medical imaging. NRRD files were included to allow for end users to quickly use 3DSlicer to view and edit segmentation in a freely available environment. NRRD masks were converted to TIFF stacks and binary labelmaps in .mat format for use in MATLAB (v. 2020b, Mathworks, Natick, MA). Muscle, bone, cartilage, ligament, and fat from the pelvis to the ankle were digitized and exported from ScanIP as raw 3D stereolithography (STL) objects without any post-processing. These raw 3D geometries may be used by others who wish to apply alternative means of post-processing than that described in steps 4 and 5.
4. The raw STL geometries were then imported into MeshMixer (v. 3.5.474, AutoDesk, Rock Hill, SC) to reduce mesh sizes, remove sharp edges, and provide a smooth geometry without substantial alteration of the original geometry. Each geometry was remeshed to the following target edge lengths: muscle 1.5 mm, bone 1.0 mm, cartilage 0.75 mm, ligament 0.75 mm. In addition, segmentation masks were generated from the processed STL files for those who wish to modify the resampled and smoothed geometries in 3D Slicer.
5. Overclosures between the resampled-smoothed 3D geometries were removed using custom MATLAB code utilizing radial basis functions^12^. All surface overclosures were removed to achieve a minimum gap distance of 0.05 mm between all geometries. Pairs of geometries that were overclosed with one of the geometries as bone, were set to remove overclosures by only deforming nodes on the mating geometry. In all other cases, overclosures were removed by deforming both geometries equally. The final geometries were exported as 3D STL objects. In addition, segmentation masks were generated for those who wish to modify the final geometries in 3D Slicer.

## Data Records

The data and metadata describing the datasets can be found at Digital Commons @ DU:^13^ 10.56902/COB.vh.2022.0 In total 260 geometries from the Visible Human Male and Female were extracted (Fig. 1)^13^. The skeletal components consist of the pelvis through the feet (Table 1); the ligament and cartilage components consist of hip, knee, and ankle cartilages and knee ligaments (Table 2); muscle components consist of 76 separate muscles from the Iliacus proximally to the Flexor Digitorum distally (Table 3), and two fat components consisting of the intramuscular fat and fascia, and the outer fat, dermis, and epidermis (Table 4).

**Table 1:** The 15 skeletal components available for download in both the female and male subjects.

	Bones of the Lower Extremities	
Coccyx	Femur × 2	Talus × 2
Sacrum	Patella × 2	Calcaneus × 2
Pelvis (*Ischium* + *Illium* + *Pubis*)×2	Tibia × 2	Navicular × 2
	Fibula × 2	Cuboid × 2
		Lateral Cuneiform × 2
		Medial Cuneiform × 2
		Intermediate Cuneiform × 2
		Phalanges (*Tarsals* + *Metatarsals* + *Phalanges*) × 2

Lower limb bones are unique on the right and left sides of the subjects (resulting in 28 unique bones per subject).

**Table 2:** The 12 identified ligaments and cartilage components available for download in both the female and male subjects.

Knee Ligaments	Articular Cartilages
Anterior Cruciate × 2	Femoroacetabular (*Femoral Head* + *Pelvis Acetabulum*) × 2
Posterior Cruciate × 2	Tibiofemoral (*Distal Femur* + *Lateral Tibia* + *Medial Tibia*) × 2
Medial Collateral × 2	Patellofemoral (*Distal Femur* + *Patellar*) × 2
Lateral Collateral × 2	Tibiotalar (*Distal Tibia* + *Proximal Talus*) × 2

Ligaments and cartilage are unique on the right and left sides of the subjects (resulting in 24 unique tissues per subject).

**Table 3:** The 38 identified muscle components available for download in both the female and male subjects.

Muscles of the Lower Extremities		
Adductor Brevis × 2	Gluteus Minimus × 2	Rectus Femoris × 2
Adductor Longus × 2	Gracilis × 2	Sartorius × 2
Adductor Magnus × 2	lliacus × 2	Semimembranosus × 2
Biceps Femoris Long × 2	lnferior Gemellus × 2	Semitendonosus × 2
Biceps Femoris Short × 2	Obturator Externus × 2	Soleus × 2
Extensor Digitorum Longus × 2	Obturator lnternus × 2	Superior Gemellus × 2
Extensor Hallucis Longus × 2	Pectineus × 2	Tensor Fasciae Latae × 2
Flexor Digitorum Longus × 2	Peroneus Longus × 2	Tibialis Anterior × 2
Flexor Hallucis Longus × 2	Piriformis × 2	Tibialis Posterior × 2
Gastrocnemius Lateral × 2	Plantaris × 2	Vastus lntermedius × 2
Gastrocnemius Medial × 2	Popliteus × 2	Vastus Lateralis × 2
Gluteus Maximus × 2	Psoas Major × 2	Vastus Medialis × 2
Gluteus Medius × 2	Quadratus Femoris × 2	

Muscle components are unique on the right and left sides of the subjects (resulting in 76 unique muscle geometries per subject).

**Table 4:** The 2 identified fat components available for download in both the female and male subjects.

Fat and Fasciae Components	
Intramuscular fat and fasciae	Outer fat (*epidermis* + *dermis* + *fat*)

Fat components are unique on the right and left sides of the subjects but contained in single files (resulting in 2 unique fat geometries per subject).

The objects available for download consist of aligned cryosection and CT images, segmentation masks of the original and processed models, raw 3D models, and processed 3D models.

- Aligned Cryosection Images: moving proximal to distal in the Visible Human sequences of cryosection images, there are offsets in the transverse plane that require correction before beginning segmentation. The Visible Human Female images contain some challenging offsets. As correction is a time-consuming process, we have made the corrected images available for download. The corrected scans are available in DICOM, TIFF, .mat, and MHD file formats.
- Aligned and Rescaled CT Images: the Visible Human CT images are useful for segmentation of tissues that are not as clear in the cryosection images. However, the original CT images are not precisely aligned with the cryosection images. We have made available the CT images aligned to the cryosection images also with offsets corrected. CT scans are full scans going from the head-to-toes. The corrected scans are available in DICOM, TIFF, and MHD file formats.
- Original Segmentation Masks: the 3D models were created using ScanIP by the construction of 3D objects from a series of outlines, or masks, of each object. This was a manual process often requiring subjective decisions when the clarity of the images made the detection of tissue borders challenging. Therefore, we have provided the segmentation masks in 3D Slicer for those that wish to verify or alter the masks for creation of unique models. The raw segmentation masks are available in 3D Slicer as NRRD files. Additionally, the segmentations are available as binary label maps in MHD, TIFF, and .mat file formats.
- Raw 3D Models: the raw 3D models created from ScanIP are provided in STL format in the default edge length of ScanIP, approximately 0.33 mm edge lengths. Providing the raw STL models enables others to apply their own preferred means of post-processing of the objects (e.g., smoothing or lofting) starting from their original state.
- Smoothed 3D Models: the smoothed and resampled STLs from MeshMixer are provided. These geometries are free of issues resulting from segmentation and each geometry was remeshed to the following target edge lengths: muscle 1.5 mm, bone 1.0 mm, cartilage 0.75 mm, ligament 0.75 mm.
- Segmentation Masks of Smoothed Models: segmentation masks were created from the smoothed and resampled 3D models to enable transverse inspection of the final product in segmentation software 3D Slicer.
- Final 3D Models: the goal of the project was to provide 3D models of the tissues that could be used in applications without further processing. The final 3D models were visually inspected to ensure no existing sharp edges and then checked and corrected for any overclosure. If an overclosure was present, it was removed to provide a gap distance of 0.05 mm using a unique radial basis function-based MATLAB code that is publicly available^12^.
- Segmentation Masks of Final Models: segmentation masks were created from the final 3D models to enable transverse inspection of the final product in segmentation software 3D Slicer.
- Comparison Metadata: includes tables of the initial overclosure amounts tissue geometries as well as comparisons between tissue volumes before and after smoothing and overclosure correction. Comparisons are also made between tissue volumes on the left and right side of the body.

The complete VHM consists of 211 GB of objects and images, and the VHF consists of 144 GB of objects and images. For this reason, the datasets have been split into manageable folders for download. The folders have been separated into those for the Male and Female, and in addition there are separate folders containing:

- Aligned Cryosection Images (DICOM)
- Aligned CT Images (DICOM)
- Aligned scans (.mat, .tif)
- Original segmentation masks and aligned scans (.mhd)
  - Right side, Left side, or combined
- Original segmentation masks (.mat, .tif)
- Original segmentation masks (3D Slicer)
  - Right side, Left side, or combined
- Segmentation masks of the smoothed 3D models (3D Slicer)
  - Right side, Left side, or combined
- Original 3D STL models
  - Right side and Left side in separate folders
- Smoothed 3D STL models
  - Right side or Left side
- Final 3D STL models
  - Right side or Left side
- Metadata

The segmentation masks are shared in a format compatible with 3D Slicer, which is publicly available at www.slicer.org. The 3D models of the tissues are shared in STL format, which is readable by most open-source and commercial 3D modeling software.

Adding new geometries of the musculoskeletal system of the lower-limb and the torso and upper limbs by other contributors is encouraged and can be added to the dataset by contacting the authors. The authors will check new or revised content for accuracy and completeness and update the folders with full credit of contributors highlighted on the website.

## Technical Validation

Creation of the segmentation maps for reconstruction of the 3D models was performed by several research assistants in the Center for Orthopaedic Biomechanics at the University of Denver. All geometries were inspected and reviewed by a panel of the authors to assure agreement with anatomy references while staying true to the original cryosection images. The anatomy references used were primarily (but not limited to) Netter^14^ and Fleckenstein *et al*.^15^, and anatomical imaging applications at Radiopedia (www.radiopaedia.org) and AnatomyLearning (www.anatomylearning.com). Primal Pictures (Informa UK Limited, London) software was used as a reference for the Visible Human Male. It is important to note that the 3D models on the left and right sides of the subjects are unique; that is, no geometries were reflected from one side to create the geometry on the contralateral side. Therefore, right-left symmetry of the models was inspected, and object volumes were compared to assure that no dramatic differences were present (e.g., the difference in muscle volume between the right and left soleus is less than 5%). For 70% of the muscles, the volume of each muscle was within 10% of the muscle on the contralateral side. Even so, we acknowledge that some inaccuracies may be present. The borders between some of the structures in the cryosection images were difficult to distinguish. Also, some disrupted anatomy in the Visible Human Female by unknown pre- or post-mortem mechanisms (e.g. left knee extensors) were segmented to provide a representative structure that appeared true to the native anatomy prior to damage. Additionally, many geometries were not included in the provided dataset, particularly the patellar tendon and complete Achille’s tendon. In this release, the primary goals were to identify bone, cartilage, muscle, and major ligaments. In recognition of these limitations, we have provided a pathway for corrective action by including contact information on the website and opportunity to contribute to updated versions of the data.

To fit the overall goal of providing simulation-ready meshes for analytical methods such as finite element analysis (FEA), custom scripts were used to remove overclosures between all pairs of geometries for each leg. 471 pairs of geometries across the Visible Human Female and the Visible Human Male were identified as having initial overclosures after the smoothing step, with more than 95% of the overclosures being between 0.1 mm and 8.8 mm, and 90% of the overclosures less than 1 mm. All overclosures were removed, and all geometries were corrected to a 0.05 mm gap distance^12^. Each geometry was inspected using custom MATLAB code to measure the final minimum distances between every pair of geometry and recorded. Verification was performed by importing geometries into Hypermesh (Altair, Troy MI) and performing the penetration and interference check with a thickness of 0.025 mm chosen. Both methods confirmed a minimum gap between geometries of 0.05 mm between any geometry. As described above, steps 4 and 5 consisted of post-processing to reduce mesh density, sharp edges, and overclosures (Fig. 2), which resulted in some small geometric change from the original raw STLs. To assure that no dramatic alterations occurred in this process, volume comparisons were made between the original and final geometries. The volumetric change was less than 15% from the original to final geometries for 95% of the structures.

## Data Availability

The final step in preparing the geometries (Step 5 in Fig. 1) utilized programming in MATLAB, which is published elsewhere^12^ and freely available open source in the GitHub repository: 10.48550/arXiv.2209.06948.In summary, a novel algorithm utilizing an iterative process of mesh reduction, over-closure/gap detection, radial basis function network (RBF) training, and nodal adjustment automatically adjusted overclosure between meshes to a desired gap distance.
